# Abdominal Wall Needle Tract Seeding: 15 Years After a Hepatocellular Carcinoma Biopsy

**DOI:** 10.7759/cureus.61131

**Published:** 2024-05-26

**Authors:** Gonçalo Saldanha, Sofia Carralas Antunes, João Cruz, Miguel Ramalho

**Affiliations:** 1 Department of Radiology, Hospital Garcia de Orta, Almada, PRT; 2 Department of Pathology, Hospital Garcia de Orta, Almada, PRT; 3 Department of Radiology, Hospital da Luz, Lisbon, PRT

**Keywords:** magnetic resonance imaging, metastasis, needle tract seeding, liver biopsy, hepatocellular carcinoma

## Abstract

Percutaneous liver biopsy, although considered a safe procedure, can lead to tumoral needle tract seeding. We describe a case of a 65-year-old woman with a history of hepatocellular carcinoma (HCC) who presented with a painless abdominal lump 15 years post-liver biopsy and left hepatectomy. An MRI revealed an abdominal wall mass suggestive of HCC metastasis from needle tract seeding. Surgical removal confirmed a well-differentiated HCC. Distinctive imaging features of HCC in specific clinical settings reduce the need for biopsy, which should be limited to exceptional cases.

## Introduction

Hepatocellular carcinoma (HCC) is the sixth most prevalent tumor globally and represents the third leading cause of cancer-related mortality worldwide [1]. The primary risk factor for developing HCC is cirrhosis, which is present in more than 80% of patients. Regardless of the underlying cause, patients with cirrhosis have an approximate 2% annual risk of developing HCC. Despite the implementation of dedicated viral hepatitis elimination programs, chronic infections with hepatitis B virus and hepatitis C virus remain the predominant etiologic risk factors in many regions [2].

Unlike most cancers, the diagnosis of HCC in at-risk patients can be confidently established using specific noninvasive imaging criteria without the need for a biopsy. According to the American Association for the Study of Liver Diseases guidelines, liver nodules exhibiting hyperenhancement on the arterial phase and washout on the portal venous or delayed phases of contrast-enhanced CT or MRI are considered diagnostic of HCC with high specificity and positive predictive value, particularly for lesions ≥2 cm in size [2]. However, in patients without liver cirrhosis or with atypical HCC imaging findings, a histopathologic diagnosis is often necessary.

In addition to diagnosis, particularly in the context of emerging molecular therapies and precision oncology, biopsies are sometimes considered for guiding treatment decisions [3]. Percutaneous liver biopsies can disrupt the tumor and the healthy surrounding tissue, increasing the probability of tumoral needle tract seeding [4]. This phenomenon, although rare, is a serious complication that may manifest several years after a percutaneous biopsy.

In this report, we present a rare case of abdominal wall implantation of HCC 15 years after percutaneous biopsy and resection of the primary lesion.

## Case presentation

A 65-year-old woman with no history of chronic liver disease underwent an abdominal ultrasound due to persistent abdominal discomfort for five weeks, which revealed a suspicious liver mass in the left lobe. A subsequent MRI scan raised suspicion for an HCC, which was confirmed by an ultrasound-guided percutaneous core needle biopsy using a noncoaxial technique. The patient underwent a left hepatectomy with an uneventful recovery and was discharged one week after the procedure. The resected specimen revealed negative surgical margins. This case was classified as stage I (pT1 pN0 pM0) according to the sixth edition of the American Joint Committee on Cancer tumor/node/metastasis classification system for HCC (edition in use at the time of diagnosis).

**Figure 1:**
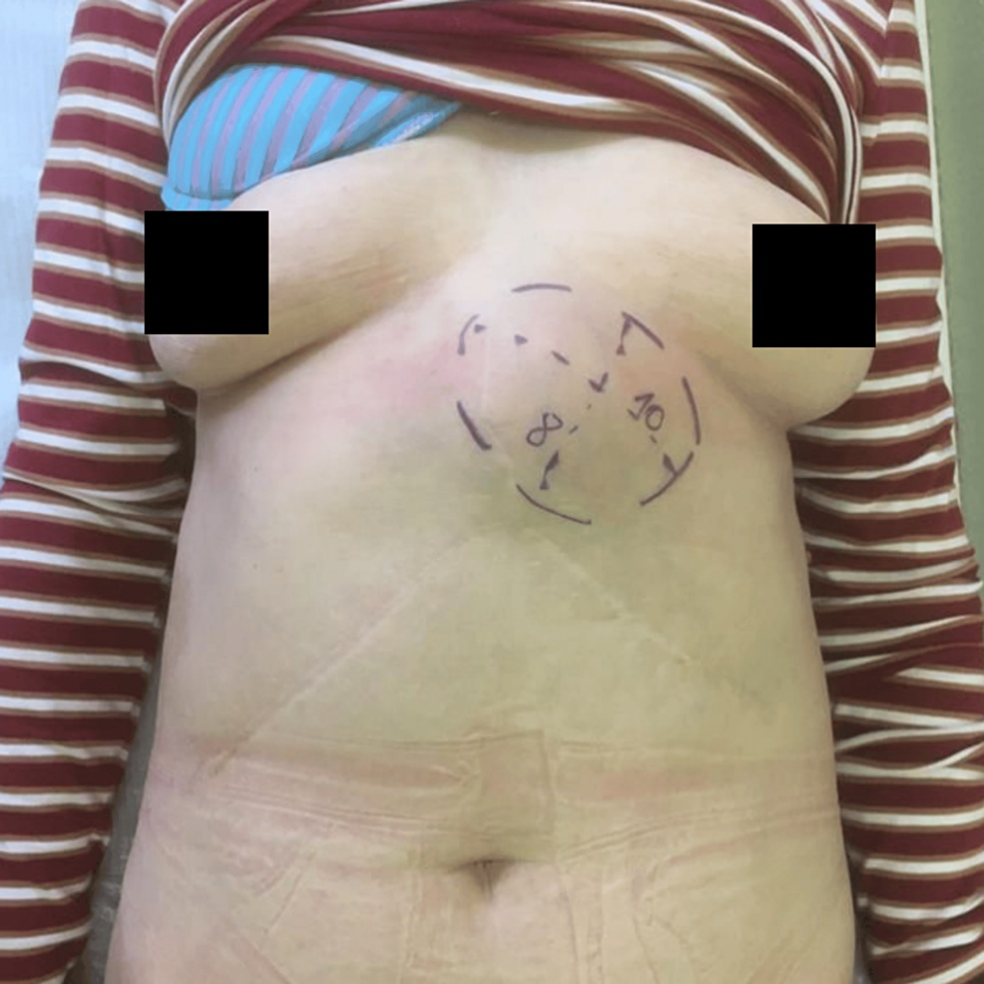
Physical examination A firm, round-shaped mass in the epigastric region (dotted marking) adjacent to the laparotomy incision.

The patient was maintained in follow-up for seven years, during which tumor markers' measurements remained consistently negative, and imaging studies (CT and MRI) revealed no evidence of tumor recurrence. About 15 years after the initial diagnosis, the patient presented to her primary care physician with a painless abdominal lump for three months without any additional symptoms reported. Physical examination revealed a firm and round-shaped mass laterally to the laparotomy incision (Figure 1).

Tumor markers showed elevated alpha-fetoprotein, carcinoembryonic antigen, and carbohydrate antigen 19-9 of 7.4 ng/mL, 9.0 ng/mL, and 32.0 ng/mL, respectively. The remaining laboratory results were unremarkable.

**Figure 2:**
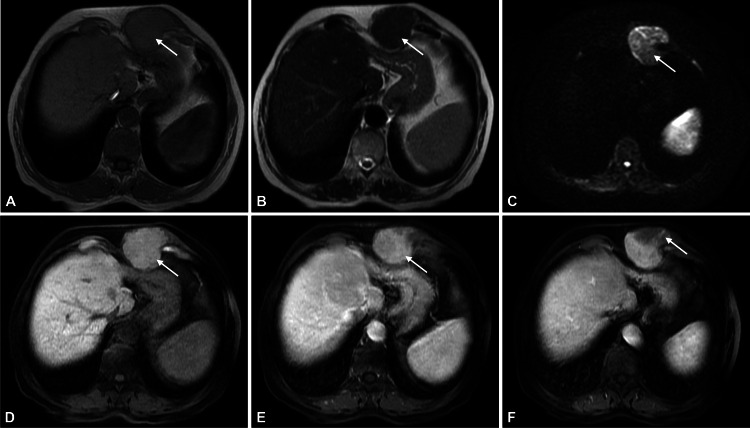
Abdominal MRI Axial T1-WI (A), axial T2-WI (B), axial DWI 600 sec/mm2 (C), axial pre- (D) and post-contrast fat-suppressed 3D-GRE T1-WI in the arterial (E) and portal-venous (F) phases. A round 6 cm mass is depicted at the anterior abdominal wall, showing T1-WI (arrow, A) and T2-WI (arrow, B) signal intensity similar to the liver parenchyma, which favors hepatocellular nature. On DWI, the mass is hyperintense on the higher gradient (b = 600 sec/mm2) (arrow, C), increasing the likelihood of malignancy. In a dynamic contrast-enhanced study, the lesion shows iso-enhancement with the liver during the arterial phase (arrow, E). However, there are small areas of washout seen in later phases (arrow, F). MRI: magnetic resonance imaging, WI: weighted images, DWI: diffusion-weighted images

An MRI scan (Figure 2) revealed a 6 cm solid mass within the anterior abdominal wall. The mass was isointense to the liver parenchyma on T1- and T2-weighted images, suggesting a hepatocellular nature, and showed comparable enhancement to the liver on the arterial phase. However, intralesional washout areas on the portal-venous and interstitial phases and restricted diffusion on diffusion-weighted images increased the likelihood of malignancy. The remaining liver showed no evidence of tumor recurrence.

**Figure 3:**
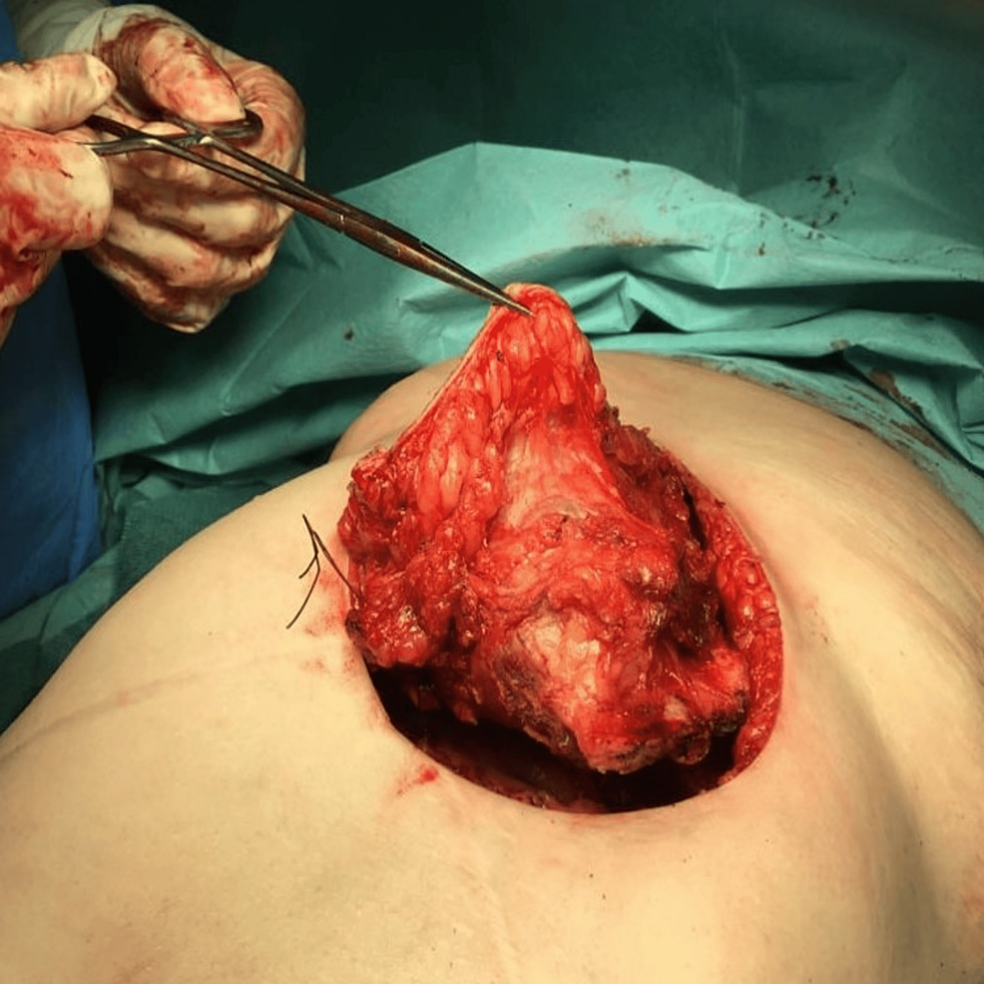
Surgical resected mass The image shows the surgically excised HCC metastasis from the anterior abdominal wall.

**Figure 4:**
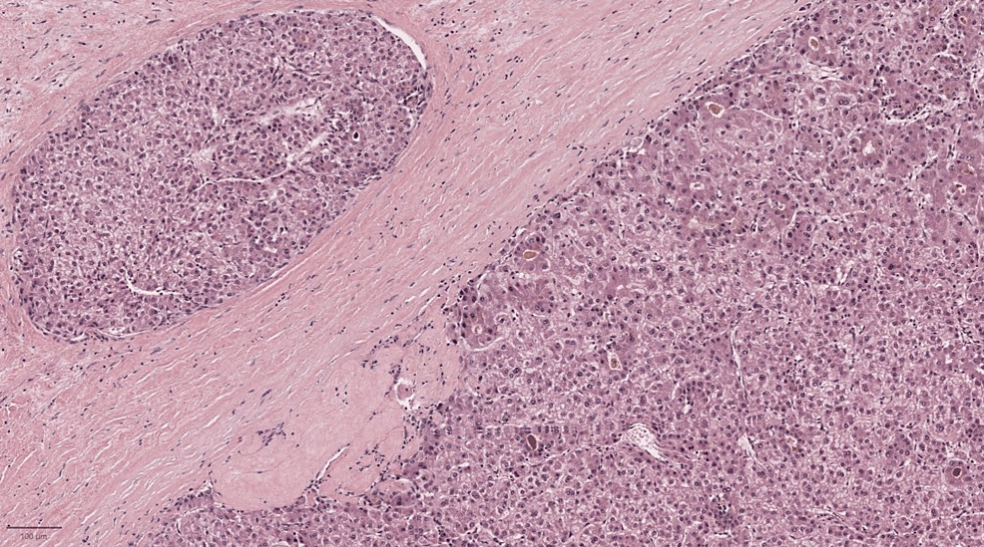
Histopathological examination Soft tissue metastasis of a well-circumscribed tumor displaying solid and pseudoglandular patterns. The tumor consists of polygonal cells with nuclear atypia and clear to eosinophilic cytoplasm, reminiscent of mature hepatocytes, leading to the diagnosis of well-differentiated HCC. HCC: hepatocellular carcinoma

The mass was biopsied, and the initial pathology report indicated focal nodular hyperplasia (FNH). However, a thorough review raised the possibility of a well-differentiated HCC. Subsequently, the mass was surgically removed (Figure 3), and the pathology report of the surgical specimen confirmed a well-differentiated HCC (Figure 4).

## Discussion

The presence of specific imaging features in an appropriate clinical context has significantly decreased the need for a liver biopsy to diagnose HCC [5]. Typically, HCC tumors exhibit distinctive radiological features attributable to their predominantly arterial supply, which becomes more evident as tumors approach or exceed 2 cm in size. Consequently, smaller tumors are less likely to have this typical radiologic appearance. Furthermore, even larger tumors may not consistently exhibit classic radiologic features, necessitating a biopsy for a definitive diagnosis [6].

Although considered a safe procedure, percutaneous liver biopsy may entail complications such as bleeding, perforation, infection, or biopsy needle tract seeding. The latter occurs when cancer cells are spread along the path of the biopsy needle, leading to the appearance of one or multiple enhancing lesions outside the liver [4].

Previous studies indicate that tumor seeding after a percutaneous liver biopsy is uncommon, with a median incidence of 2.29% [4]. Presumed risk factors for needle tract seeding include tumor location and aggressiveness, biopsy technique, needle size, number of needle passes, and patient immunosuppression.

The coaxial biopsy technique, whereby the biopsy needle is guided through an introducing needle, permits the extraction of multiple samples from the lesion with a single puncture. This enclosed system method also helps protect normal tissue along the biopsy needle tract. Maturen et al. [7] reviewed the incidence of needle tract seeding after 101 percutaneous liver biopsies for HCC using exclusively a coaxial technique. They reported no cases of tumor seeding in this patient cohort during a six-year follow-up period. Despite these favorable results, a randomized trial comparing noncoaxial and coaxial needle techniques is required to provide more compelling evidence. The optimal needle diameter and number of needle passes to minimize the risk of seeding are also suggested as important variables [8]. However, there is scarce published literature investigating these aspects, and further research is needed to establish more robust evidence.

The average time for detecting needle tract seeding varies widely across different studies, ranging from a few months to 13 years [9-11]. Consequently, researchers experience significant challenges regarding the necessary duration of follow-up after a biopsy, potentially resulting in the underreporting of tumor seeding occurrences. In our case, tumor seeding was observed 15 years after the liver biopsy. To the best of our knowledge, there are no documented cases in the literature with such an exceedingly long delay. This may be partially related to the slow-growing behavior of this well-differentiated tumor. However, we cannot be certain as there are also reports of well-differentiated tumors detected within a year after biopsy [12,13].

The presence of a mass near the presumed biopsy needle path and its appearance on MRI raised suspicion for HCC metastatic seeding. Upon biopsy, the initial pathology report indicated FNH instead of HCC. This misdiagnosis is attributed to the challenges of histologically differentiating between well-differentiated HCC and benign liver nodules [14].

## Conclusions

This case report presents the longest recorded time for diagnosing HCC needle tract seeding, raising awareness that seeding can still occur several years after a percutaneous biopsy. Hence, we emphasize limiting liver biopsies to exceptional cases and ensuring proper follow-up after effective treatment.
